# A population-based study of effect of multiple birth on infant mortality in Nigeria

**DOI:** 10.1186/1471-2393-8-41

**Published:** 2008-09-10

**Authors:** Olalekan A Uthman, Mubashir B Uthman, Ismail Yahaya

**Affiliations:** 1 Center for Evidence-Based Global Health, Ilorin, Kwara State, Nigeria; 2 Department of Epidemiology and Community Health, University of Ilorin, Ilorin, Kwara State, Nigeria

## Abstract

**Background:**

Multi-foetal pregnancies and multiple births including twins and higher order multiples births such as triplets and quadruplets are high-risk pregnancy and birth. These high-risk groups contribute to the higher rate of childhood mortality especially during early period of life.

**Methods:**

We examined the relationship between multiple births and infant mortality using univariable and multivariable survival regression procedure with Weibull hazard function, controlling for child's sex, birth order, prenatal care, delivery assistance; mother's age at child birth, nutritional status, education level; household living conditions and several other risk factors.

**Results:**

Children born multiple births were more than twice as likely to die during infancy as infants born singleton (hazard ratio = 2.19; 95% confidence interval: 1.50, 3.19) holding other factors constant. Maternal education and household asset index were associated with lower risk of infant mortality.

**Conclusion:**

Multiple births are strongly negatively associated with infant survival in Nigeria independent of other risk factors. Mother's education played a protective role against infant death. This evidence suggests that improving maternal education may be key to improving child survival in Nigeria. A well-educated mother has a better chance of satisfying important factors that can improve infant survival: the quality of infant feeding, general care, household sanitation, and adequate use of preventive and curative health services.

## Background

Despite significant improvements in child survival in recent decades, levels of infant and child mortality and morbidity remain unacceptably high in many developing countries [1,2]. These problems are particularly serious among high-risk pregnancies and births, and in developing countries where the health-care system is still struggling to provide basic public health and maternal and child health care to their population[3,4]. In such countries, adequate health-care services for managing high-risk pregnancy and delivery are usually available at the referral levels such as regional and national hospitals[5,6]. However, access to these facilities remain limited owing to factors such as distance, transportation cost and medical fees; specifically for the poor women and women who live in the rural and remote areas[7].

Multiple births are relatively rare events, but contribute substantially to mortality in both neonatal and post-neonatal periods[8]. Yoruba of western Nigeria is considered "land of twins". Almost 5 percent of all Yoruba births produce twin, compared with just around 1.2 percent for Western Europe and 0.8 percent for Japan. Previous studies have associated high infant mortality with multiple births in both developing [7-14] and developed [15-22] countries; specifically those with birth defects, premature birth and low birth-weight. To the best of our knowledge no study has examined the effects of multiple births on infant mortality in Nigeria. Therefore, the aim of this study was to examine if infants of multiple births have disproportionately higher risk of mortality than infants of singleton births.

## Methods

### Data source

This study uses data from the 2003 Nigeria Demographic and Health Survey (NDHS) [23]. It is based on information of 6219 children born within five years prior to the survey. The NDHS collected demographic, socio-economic, and health data from nationally representative sample of 7620 women aged 15–49 years in 7864 households included in the survey. The state was stratified into 36 states and the Federal Capital Territory (FCT) of Abuja within the six geopolitical regions. Methods used in the NDHS have been published elsewhere [24].

Briefly, each domain is made up of enumeration areas (EAs) established by a general population and housing census in 1991. The sampling frame was a list of all EAs (clusters). Within each domain, a two-stage sample was selected. The first stage involved selecting 466 clusters (primary sampling units) with a probability proportional to the size, the size being the number of households in the cluster. The second stage involved the systematic sampling of households from the selected clusters.

### Ethical consideration

This study is based on an analysis of existing survey data with all identifier information removed. The survey was approved by the Ethics Committee of the ORC Macro at Calverton in the USA and by the National Ethics Committee in the Ministry of Health in Nigeria. All study participants gave informed consent before participation and all information was collected confidentially.

### Variables

#### Outcome variable

Each woman interviewed in the survey was asked to provide a detailed history of all her live births in chronological order, including whether a birth was single or multiple, sex of the child, date of birth, survival status, age of the child on the date of interview if alive, and if not alive, age at death of each live birth. These data from the birth histories were used to calculate infant mortality rate, defined as the probability of dying before completing 12 months of age, using a synthetic cohort life table[25]. The rate was expressed as deaths per 1000 live births.

#### Exposure variable

The multiple birth status was analysed as not multiple-birth (singleton) and multiple-birth (twin, triplet, quadruplet, or higher order). Each multiple birth child was analysed as an individual child, and the clustering effect of each group of multiple births was included in the analysis.

#### Potential confounders

Because child survival is correlated with pregnancy care, delivery assistance, maternal nutrition, household living conditions, and other child, mother, and household characteristics and socio-economic factors that can also affect morbidity and mortality in children, the association between multiple birth status and infant mortality were estimated after adjusting for the effects of these other risk factors and potentially confounding factors. These factors include child's sex (boy, girl), professional assistance at delivery (no, yes), birth order (1, 2, 3, 4+), child's birth size (below average, average, above average), mother's age at childbirth (13–17, 18–24, 25–34, 35–48), mother's body mass index (BMI) (<18.5, 18.5–24.9, 25.0+ kg/m^2^), mother's education (no education, some primary, secondary or higher), household wealth index (highest, fourth, middle, second, lowest), household access to safe drinking water (yes, no), availability of a hygienic toilet (yes, no), cooking fuel type (low pollution fuel, high pollution fuel), ethnic group (Hausa/Fulania, Igbo, Yoruba, others) residence (urban, rural) and geographic division (North central, North East, North West, South East, South South, and South West).

### Statistical analysis

We used univariable and multivariable survival regression procedure with Weibull hazard function in Stata version 10[26] to examine the relationship of multiple birth status and other factors on infant mortality. A number of unadjusted hazard regression models were used to assess the unadjusted effect of multiple births and different risk factor and confounding factor, and a full adjusted model to assess the adjusted effect of multiple births controlling for all other factors that were significant in the unadjusted analyses (p < .05). In our analysis, weights were used to restore the representativeness of the sample, in which certain categories of respondents were over-sampled and non-response rates varied from one geographical area to another. Results were presented as hazard ratios (HR) with 95% confidence intervals (CI).

## Results

The sample distribution of children born in the five years preceding the 2003 Nigeria Demographic and Health Survey (NDHS) by multiple birth status and other selected characteristics are shown in Table 1 (see additional file 1). About 4% of children are of multiple births. Fifty-one percent of all births were boys and 49% were girls.

About one third received professional assistance at delivery. Twenty-one per cent of children were first order births, and 49% were fourth or higher order births. Only 9% of the births were to mothers aged 13–17, about 36% and 40% were to mothers' aged 18 – 24 and 25–34 years respectively, and the remaining 14% to mothers aged 35–48 years. Only 12% of the births were to undernourished mothers (BMI < 18.5 kg/m^2^), more than half (51%) of the births were to illiterate mothers. Consistent with higher fertility in the poorer households, 44% of children were born in the poorest 40% households, and 17% were born in the richest 20% households. Only (26%) of children were born in households with safe sources of drinking water. Most of the children (87%) were in households without a hygienic toilet facility. Similarly, most of the children (79%) were in households using high pollution fuels (firewood or straw) for cooking. Seventy-one per cent of births were in rural areas. By geographic division, more than one-third (35%) of the births were in North West, 24% were in North East, 14% were in North Central, 13% were in South South, 9% were in South West, and only 6% were in South East

On average, more than one in every 10 children born in Nigeria (101 per 1000 live births) does not survive to their first birthday. The infant mortality rate was very high among the multiple birth children – 236 per 1000 live births compared with 95 per 1000 live births among the singletons. The infant mortality rate was also higher among boys (107) than among girls (95). Mothers who received professional assistance at delivery were associated with lower infant mortality rate. There was a U-shaped relationship between infant mortality rate and childbirth order. Children were born to the older mothers (35–48 years old) were at greater risk of infant mortality. As expected infant mortality rate was strongly negatively associated with mother's educational status.

The probability of death before 12 months of age for children born in the poorest 20% households was greater than children born in the richest 20% households. Households that lacks of a hygienic toilet facility were associated with higher risk of infant mortality. Similarly, non-availability of safe drinking water in households was associated with higher risk of infant mortality. Infant mortality rate was higher in rural area than in urban area; and it was considerable higher in North East than other geographical divisions.

### Association of multiple birth and infant survival

**Figure 1:**
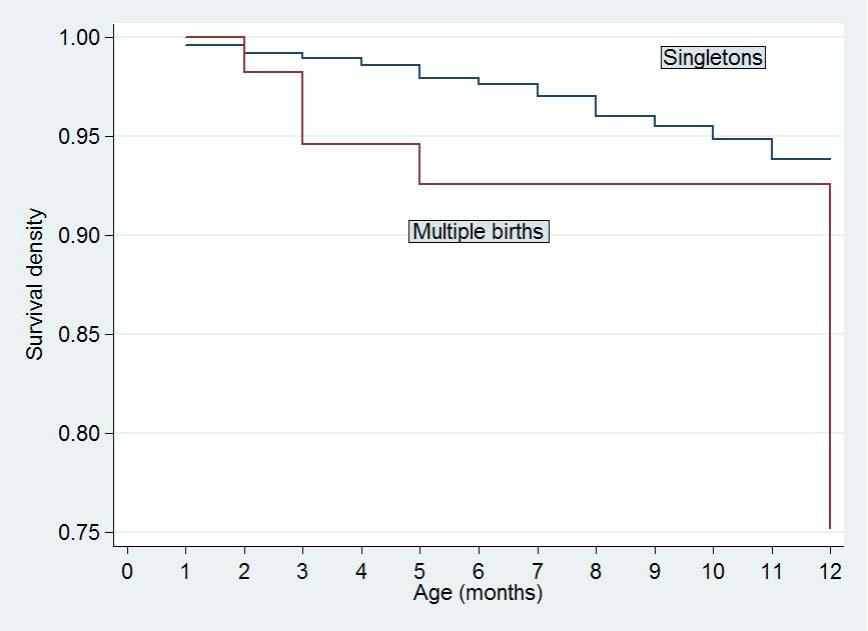
Probability of survival before 12 months of age by birth status, Nigeria 2003.

For children born in the five years preceding the NDHS, the survival probabilities for children born singleton and multiple-birth by single months of age before 12 months was presented in Figure 1. The figure shows that the two survival curves diverge beyond the second month of age, with children born multiple-birth having progressively lower survival probability than children born singleton. The difference in survival probability between children born singleton and multiple-birth was non-random as judge by log-rank test of survival functions (chi-squared = 16.01 on one degree of freedom; p = .001). The large majority of multiple birth children died in infancy.

The unadjusted hazard ratio for the effect of multiple births indicates that there was a strong positive relationship between multiple-birth status and infant mortality (Table 2 – see additional file 2). Children born multiple-birth were about two times as likely to die in infancy as those born singleton (hazard ratio [HR] = 2.00; 95% confidence interval [CI]: 1.43, 2.81). The Unadjusted models show that the infant mortality was significantly associated with delivery by a health-care professional, mother's BMI, mother's education, safe source of drinking water, hygienic toilet facility, type of fuel used for cooking, urban/rural residence and geographic division. In the adjusted model, when all characteristics of the child and mother, household wealth status, availability of safe drinking water, availability of a hygienic toilet facility, type of fuel used for cooking, urban/rural residence and geographic divisions were controlled for, the relationship of multiple birth and infant mortality remains significant. With all child-, maternal-, household-specific and other factors controlled, children born multiple birth were more than twice as likely to die during infancy as infants born singleton (HR = 2.19; 95% CI: 1.50, 3.19).

### Effects of other risk factors and confounders on infant survival

In the adjusted model (Table 2 – see additional file 2), with multiple birth status and other factors controlled, children born to mothers with secondary education or more were less likely to die before reaching their first birthday than children of mothers with no education (HR = 0.51; 95% CI: 0.36, 0.72). Compared to children from the poorest households, infant from the richer households are less likely to die before their first birthday (HR 0.67; 95% CI: 0.47, 0.96). None of the other risk factors or confounders has a statistically significant effect on the risk of infant mortality.

## Discussion

Results of this study support claims that children born multiple births are more likely to die during the first year of life as children born singletons, independent of child's sex, birth order, pregnancy care and delivery care, maternal education and nutritional status, household access to clean water and sanitation, and other factors. It is important to note that the more fully adjusted model showed a higher estimated probability of children born multiple births not to survive beyond their first birthday than the unadjusted model. This illustrate that the adjusted model was more effective in predicting the risk of infant mortality among children born multiple births, which is supported theoretically[27]. The association between multiple birth and infant mortality has been observed not only in developing countries [7-14] but also in developed countries [15-22].

One possible reason for this observed association is that multi-foetal pregnancy and multiple births including twins and higher order multiples such as triplets and quadruplets are high-risk pregnancy and birth. These high-risk births are frequently accompanied by a number of associated foetal and neonatal complications that require special and expensive medical care [28]. In addition, multiple-birth children are at much greater risk of birth defects and/or disabilities and accounted for larger percentage of prenatal deaths [29]. Therefore, mortality of these high-risk groups contributes to the higher rate of childhood mortality especially during the early period of life. We also found that large majority of multiple birth children died in infancy. This is consistent with finding from previous study[11]. The perinatal period has long been recognized as a period of increased risk for twins. [11]. It is possible that the use of the curative services, which are needed in emergency situations, could be under-utilized by mothers of twins[11]. However, the increased mortality risk extends into the post-neonatal and childhood period, when the mortality of twins is more than twice the value of the mortality of singletons

Consistent with previous studies [30-34], we found that mother's education has strong negative effect on infant mortality, independent of other factors. There is a very large literature from around the world that demonstrates the significance of mother's schooling to lower mortality outcome among children. The pathways from mother's schooling lower mortality rate include, but are not limited to, greater likelihood of obtaining pre- and ante-natal care, seeking prompt medical care at the first sign of a child's illness, and more appropriate breast feeding and nutritional supplementary practices. This and previous studies [14,35-40]have provided evidence that children born in the poorer households are a much greater risk of dying in infancy than children born in better-off households. Poverty affects infant survival through insufficient food intake, greater exposure to infections, and lack of access to vaccinations and basic health care.

### Study limitations and strengths

Finally, it is necessary to discuss the limitations of this study, as well as its strengths. The present study was performed in a large nationally representative sample with stratified random sampling. The first potential limitation of this study is the cross-sectional nature of the analysis. The study uses censored, synthetic cohort life table based on the birth history of children and reported characteristics of mothers and households. While some of the covariates used in our analysis, such as child's sex, antenatal care, mother's age at childbirth are fixed covariates, others, such as mother's education, household access to clean water and sanitation, and urban/rural residence could have changed during the last 5 years. In the analysis, all covariates are assumed to have been fixed during the study period. However, because the household characteristics and many of the other background characteristics are not likely to have changed much in the past 5 years and because the main finding in the analysis is relationship of multiple birth status and child survival, the cross-sectional effects estimated in these studies are good measures of child survival among multiple births.

Another limitation of this study worth mentioning is that measuring wealth is problematic. Many of the household wealth indices use assets that are more likely to be found in urban areas than in rural areas. Thus, most of the rural households will be in the lowest wealth category even if they have other indicators of wealth (e.g., livestock or farm machinery). The consequence of this misclassification would be to lower the mortality risks of rural households. Another limitation with household wealth indices derived from DHS is that they are based on current status data so that they might not capture the true level of household wealth during the infancy of children born several years before the survey. However, since these analyses are restricted to births within five years of the surveys, this bias will not be substantial.

## Conclusion

In summary, we found that children born multiple births are more likely to die during the first year of life compare to children born singletons, independent of child's sex, birth order, pregnancy care and delivery care, maternal education and nutritional status, household access to clean water and sanitation, and other factors. We also found that the mother's education played a protective role against infant death. This evidence suggests that improving maternal education may be key to improving child survival in Nigeria. A well educated has a better chance of satisfying important factors that can improve infant survival: the quality of infant feeding, general care, household sanitation, and adequate use of preventive and curative health services.

## Competing interests

The authors declare that they have no competing interests.

## Authors' contributions

OAU: Major role in study conception, data extraction, analyses, and writing of the manuscript. MBU: Data extraction, statistical analyses of data and manuscript writing. IY: Data extraction, statistical analyses of data and manuscript writing. All authors have read and approved the final manuscript.

## Pre-publication history

The pre-publication history for this paper can be accessed here:



## Supplementary Material

Additional file 1 Table 1. Sample distribution and infant mortality rate (IMR) among children born during 1999 – 2003 by household wealth status and other selected characteristics, Nigeria 2003*. Click here for file

Additional file 2 Table 2. Unadjusted and adjusted hazard ratio estimates the risk of multiple-birth mortality before 12 months of age, controlling for several factors among children born during 1999 – 2003, Nigeria 2003^‡^. Click here for file
