# Supplementary material for: A population-based study of effect of multiple birth on infant mortality in Nigeria
Source: BMC Pregnancy Childbirth. 2008 Sep 10;8:41. doi: 10.1186/1471-2393-8-41 (PMC2551580; doi:10.1186/1471-2393-8-41)
Supplement: Additional file 1 — Table 1. Sample distribution and infant mortality rate (IMR) among children born during 1999 – 2003 by household wealth status and other selected characteristics, Nigeria 2003*. [file 1471-2393-8-41-S1.doc]

**Table 1: Sample distribution and infant mortality rate (IMR) among children born during 1999 – 2003 by household wealth status and other selected characteristics, Nigeria 2003^*^**

| **Characteristics** | **Weighted sample** | **Sample distribution (%)^**^** | **IMR per 1000 Live births** |
| --- | --- | --- | --- |
| **Nigeria** | 6219 |  | 100.8 |
| **Child of multiple birth** |  |  |  |
| No | 5971 | 96 | 95.0 |
| Yes | 248 | 4 | 235.8 |
| **Child’s sex** |  |  |  |
| Girl | 3033 | 48.8 | 94.9 |
| Boy | 3186 | 51.2 | 106.6 |
| **Delivery by a health care professional** |  |  |  |
| No | 4151 | 66.7 | 109.3 |
| Yes | 2025 | 32.6 | 73.0 |
| **Child’s birth size** |  |  |  |
| Small size | 904 | 14.5 | 137.9 |
| Average size | 2548 | 41.0 | 92.9 |
| Large size | 2686 | 43.2 | 58.9 |
| **Child’s birth order** |  |  |  |
| 1 | 1278 | 20.6 | 115.6 |
| 2 | 1001 | 16.1 | 76.5 |
| 3 | 907 | 14.6 | 89.7 |
| 4+ | 3032 | 48.8 | 106.5 |
| **Mother’s age at childbirth** |  |  |  |
| 13 – 17 | 551 | 8.9 | 132.8 |
| 18 – 24 | 2270 | 36.5 | 100.0 |
| 25 – 34 | 2505 | 40.3 | 91.2 |
| 35 – 48 | 892 | 14.3 | 110.8 |
| **Mother’s BMI (kg/m^2^)** |  |  |  |
| 18.5 – 24.9 | 4004 | 64.4 | 104.8 |
| < 18.5 | 773 | 12.4 | 91.7 |
| ≥ 25 | 1305 | 21.0 | 91.8 |
| **Mother’s education level** |  |  |  |
| No education | 3224 | 51.8 | 115.3 |
| Primary | 1465 | 23.6 | 108.4 |
| Secondary or higher | 1530 | 24.6 | 64.6 |
| **Household economic status** |  |  |  |
| Poorest | 1394 | 22.4 | 128.7 |
| Poor | 1379 | 22.2 | 136.5 |
| Rich | 1255 | 20.2 | 96.5 |
| Richer | 1157 | 18.6 | 70.1 |
| Richest | 1033 | 16.6 | 53.3 |
| **Hygienic toilet** |  |  |  |
| No | 5436 | 87.4 | 107.7 |
| Yes | 782 | 12.6 | 50.0 |
| **Safe water source** |  |  |  |
| No | 4597 | 73.9 | 107.2 |
| Yes | 1620 | 26.1 | 83.1 |
| **Cooking fuel** |  |  |  |
| Low pollution fuel | 4934 | 79.3 | 107.0 |
| High pollution fuel | 1174 | 18.9 | 68.9 |
| **Type of residence** |  |  |  |
| Urban | 1795 | 28.9 | 75.6 |
| Rural | 4424 | 71.1 | 114.5 |
| **Ethnicity** |  |  |  |
| Hausa / Fulani | 2708 | 43.5 | 107.6 |
| Igbo | 573 | 9.2 | 96.3 |
| Yoruba | 490 | 7.9 | 58.3 |
| Others | 2425 | 39.0 | 105.4 |
| **Region** |  |  |  |
| North central | 897 | 14.4 | 93.5 |
| North east | 1472 | 23.7 | 110.6 |
| North west | 2161 | 34.8 | 108.0 |
| South east | 371 | 6.0 | 96.8 |
| South south | 789 | 12.7 | 107.1 |
| South west | 529 | 8.5 | 65.8 |

*Data source: Nigeria 2003 Demographic and Health Survey (n=6029)[23]

**Percentages may not add up to 100% because of missing values
