# Supplementary material for: A population-based study of effect of multiple birth on infant mortality in Nigeria
Source: BMC Pregnancy Childbirth. 2008 Sep 10;8:41. doi: 10.1186/1471-2393-8-41 (PMC2551580; doi:10.1186/1471-2393-8-41)
Supplement: Additional file 2 — Table 2. Unadjusted and adjusted hazard ratio estimates the risk of multiple-birth mortality before 12 months of age, controlling for several factors among children born during 1999 – 2003, Nigeria 2003‡. [file 1471-2393-8-41-S2.doc]

**Table 2: Unadjusted and adjusted hazard ratio estimates the risk of multiple-birth mortality before 12 months of age, controlling for several factors among children born during 1999 – 2003, Nigeria 2003**‡

|  | **Unadjusted model** | **Adjusted model** |
| --- | --- | --- |
|  | **HR (95% CI)** | **HR (95% CI)** |
| **Child of multiple birth^‡‡^** |  |  |
| No^†^ | 1 | 1 |
| Yes | 2.00 (1.43, 2.81) **^***^** | 2.19 (1.50, 3.19)**^***^** |
| **Child’s sex** |  |  |
| Girl^†^ | 1 |  |
| Boy | 0.89 (0.76, 1.06) | ni |
| **Delivery by a health care professional** |  |  |
| No^†^ | 1 | 1 |
| Yes | 0.46 (0.37, 0.57)**^***^** | 0.85 (0.65, 1.12) |
| **Child’s birth size^‡‡‡^** |  |  |
| Small size | 1.47 (1.16, 1.88)**^**^** | ni |
| Average size | 1 | ni |
| Large size | 0.96 (0.79, 1.17) | ni |
| **Child’s birth order** |  |  |
| 1^†^ | 1 |  |
| 2 | 0.86 (0.64, 1.15) | ni |
| 3 | 1.03 (0.77, 1.37) | ni |
| 4+ | 1.12 (0.90, 1.41) | ni |
| **Mother’s age at childbirth** |  |  |
| 13 – 17^†^ | 1.33 (1.00, 1.77) |  |
| 18 – 24^†^ | 1 |  |
| 25 – 34 | 0.89 (0.73, 1.09) | ni |
| 35 – 48 | 1.19 (0.93, 1.52) | ni |
| **Mother’s BMI (kg/m^2^)** |  |  |
| 18.5 – 24.9^†^ | 1 | 1 |
| < 18.5 | 0.96 (0.74, 1.24) | 0.89 (0.68, 1.17) |
| ≥ 25 | 0.70 (0.55, 0.88)**^**^** | 0.97 (0.75, 1.26) |
| **Mother’s education level** |  |  |
| No education^†^ | 1 | 1 |
| Primary | 0.80 (0.66, 0.98)**^*^** | 0.91 (0.71, 1.15) |
| Secondary or higher | 0.36 (0.28, 0.47)**^***^** | 0.51 (0.36, 0.72)**^***^** |
| **Household economic status** |  |  |
| Poorest^†^ | 1 | 1 |
| Poor | 1.03 (0.83, 1.28) | 1.02 (0.80, 1.30) |
| Rich | 0.75 (0.59, 0.96)**^*^** | 0.87 (0.67, 1.14) |
| Richer | 0.52 (0.40, 0.69)**^***^** | 0.67 (0.47, 0.96)**^*^** |
| Richest | 0.34 (0.25, 0.48)**^***^** | 0.86 (0.45, 1.64) |
| **Hygienic toilet** |  |  |
| No^†^ | 1 | 1 |
| Yes | 0.36 (0.25, 0.53)**^***^** | 0.63 (0.38, 1.06) |
| **Safe water source** |  |  |
| No^†^ | 1 | 1 |
| Yes | 0.66 (0.53, 0.81)**^***^** | 0.92 (0.71, 1.17) |
| **Cooking fuel** |  |  |
| High pollution fuel^†^ | 1 | 1 |
| Low pollution fuel | 0.44 (0.33, 0.59)**^***^** | 1.19 (0.70, 2.02) |
| **Type of residence** |  |  |
| Urban ^†^ | 1 | 1 |
| Rural | 1.74 (1.43, 2.12)**^***^** | 1.14 (0.88, 1.49) |
| **Ethnicity** |  |  |
| Hausa / Fulani | 1 | 1 |
| Igbo | 0.42 (0.30, 0.60)**^***^** | 0.64 (0.29, 1.42) |
| Yoruba | 0.40 (0.27, 0.58)**^***^** | 0.92 (0.51, 1.64) |
| Others | 0.79 (0.66, 0.94)**^**^** | 0.95 (0.72, 1.24) |
| **Region** |  |  |
| North central^†^ | 1 | 1 |
| North east | 1.50 (1.15, 1.96)**^**^** | 1.23 (0.90, 1.67) |
| North west | 1.49 (1.15, 1.93)**^***^** | 1.12 (0.79, 1.59) |
| South east | 0.72 (0.47, 1.11) | 0.59 (0.23, 1.49) |
| South south | 1.10 (0.76, 1.58) | 1.45 (0.99, 2.13) |
| South west | 0.60 (0.39, 0.92)**^*^** | 0.74 (0.44, 1.22) |

HR – hazard ratio, CI – confidence interval, ni – not included

**^‡^**Data source: Nigeria 2003 Demographic and Health Survey (n=6029)[23]

**^‡‡^**Standard errors were adjusted for clustering effect of each group of multiple, relaxing the usual requirement that the observations be independent.

**^‡‡‡^**Birth size was not included in the adjusted model due to endogeneity with multiple births which creates significant multicollinearity

^†^Reference category

**^*^**p<.05, **^**^**p<.01, and **^***^**p<.001
